# Perceptions and Misperceptions of Early Palliative Care Interventions for Patients With Hematologic Malignancies Undergoing Bone Marrow Transplantation

**DOI:** 10.7759/cureus.13876

**Published:** 2021-03-14

**Authors:** Corey Suthumphong, Dan B Tran, Marco Ruiz

**Affiliations:** 1 Translational Medicine, Florida International University, Herbert Wertheim College of Medicine, Miami, USA; 2 Miami Cancer Institute, Baptist Health South Florida, Miami, USA

**Keywords:** supportive and palliative care, hematologic malignancies, bone marrow transplantation

## Abstract

Hematopoietic stem cell or bone marrow transplantation (BMT) is one of the most promising and potentially curative therapeutic options available for eligible patients with hematologic malignancies (HMs) or leukemias. However, the nature and clinical course of HMs, specifically for patients undergoing BMT, are associated with significant morbidity, symptomatology, healthcare service utilization, psychosocial and end of life issues, and overall decreased quality of life. Early palliative care (PC) consultations and utilization for patients with HMs have been shown to improve patient outcomes, satisfaction, and autonomy as well as caregiver burden, shared-decision making, and holistic care management. Despite the complexity of care and complications for patients with HM undergoing BMT, early PC interventions are systematically underutilized and understudied in this population compared to patients with solid tumors or non-HMs. Herein, the authors reviewed the current literature and knowledge to assess and report the perceptions and barriers to early PC utilization in the care of patients with HMs undergoing BMT. Clinical and cultural aspects of PC perceptions as well as current PC care models and potential directions for PC implementation were reviewed to inform future research studies and clinical practice guidelines necessary for the improvement of care and quality of life for HM patients undergoing BMT.

## Introduction and background

Hematologic malignancies (HMs), or leukemias, are a common form of cancer in the United States, with over 60,000 estimated new cases in 2019 and over 400,000 individuals living with a form of leukemia in 2016 [1]. However, when compared to patients with solid tumors and malignancies, multiple studies across several centers have found that patients with HMs receive suboptimal care near the end of life [2]. Patients with HMs are more likely to experience a lower quality of life through higher rates of emergency room (ER) visits, hospital or intensive care unit (ICU) admissions, hospital or ICU deaths, chemotherapy within the last 14-30 days of life, and aggressive care at the end of life. Importantly, patients with HMs compared to solid tumors are significantly less likely to receive specialty palliative care (PC) interventions or consultations and die in hospice care [2]. Previous studies have shown that early PC interventions in the oncologic patient population are associated with increased patient agency and care participation, improved quality of life, mood, satisfaction of care, and caregiver outcomes, as well as reductions in aggressive care at the end of life [3]. PC interventions allow for establishing meaningful provider relationships earlier in the course of treatment and for facilitating goals of care or advanced care planning discussions that may help reduce aggressive treatment near the end of life and improve overall quality and satisfaction of care. However, current guidelines in the National Comprehensive Cancer Network have not yet incorporated PC interventions for patients with HMs. Furthermore, this remains an understudied topic that needs greater insight as a majority of randomized clinical trials assessing the benefits and effects of PC interventions often do not include patients with HMs [3]. Yet, some single-center studies both in the United States and in Taiwan analyzing effects of early PC interventions for patients with HMs have demonstrated significant reductions in ICU admission, chemotherapy usage, inappropriate cancer treatment, and cardiopulmonary resuscitation (CPR) necessity in the final month of life [4]. A better understanding of the perceptions and barriers of early PC interventions in the HM patient population is needed to begin implementing PC into standard care practice to improve the quality of life for HM patients throughout their healthcare experience.

 For patients with HMs, one of the most important potential treatment options is hematopoietic cell transplantation, also known as bone marrow transplantation (BMT). BMT potentially promotes remission or even a cure for patients with HMs and provides the greatest probability of long-term survival for these individuals [5]. Approximately 22,000 patients receive a BMT in the United States every year. However, the subpopulation of patients with HMs who receive a BMT are the most likely to receive aggressive care at the end of life due to the numerous complications and symptomatology pre-, peri-, and post-transplantation. Common symptoms include severe nausea, vomiting, and diarrhea, and common complications from treatment include drug toxicity, bleeding, infection, mucositis, graft rejection, graft-versus-host disease, and severe cardiac and/or respiratory complications [2]. Patients undergoing BMT have intense emotional, psychosocial, and spiritual issues and considerations arising around the time of treatment [6]. Early PC interventions are known to provide critical advice for symptom management and control throughout the patient care experience while emphasizing goals of care and establishing advanced care decisions to improve the quality of care according to patient preferences, values, and decisions. Thus, early PC interventions may be of great benefit for HM patients undergoing BMT and aid in the important and difficult transition points from curative to PC when necessary. Through improving physical and emotional symptom control, timely PC interventions may also prevent medical crises and possibly lower rates of ER visits, hospitalizations, and aggressive treatment for symptom control near the end of life for these patients. However, there are limited numbers of studies analyzing the effects and benefits of early PC interventions for patients with HMs undergoing BMT [7]. Furthermore, the intensive care and monitoring required during the first few years post-BMT to manage symptoms, complications, morbidity, and mortality emphasize the need for a better understanding of barriers and perceptions regarding early PC interventions for this patient population. Improving this understanding can inform future studies and models on the necessary considerations for implementing PC for HM patients undergoing BMT to improve health outcomes and maximize quality of life and care.

## Review

Clinical barriers and disparities

 While select studies have shown that implementing PC interventions early on in the care of patients with HMs undergoing BMT has numerous positive outcomes and effects, important clinical barriers and disparities have also been identified in these care models [4]. These different barriers and challenges for early PC interventions can be divided into clinical or provider barriers, patient barriers, and logistical or administrative barriers. One of the most common involves the difficulty of prognostication and optimistic perspectives toward the assessment of patient outcomes. Several studies have demonstrated that both providers and patients tend to overestimate prognosis and patient survival times [6]. Because of the uncertainty of an exact prognosis and estimated recovery, this challenges and introduces hesitancy toward the providers’ and patients’ shared decision on when to begin facilitating advanced-care discussions and incorporating PC services. Furthermore, a major challenge revolves around both the providers’ and patients’ hesitancy to discuss the transition point from curative treatment to palliative treatment. For patients with HMs undergoing BMT specifically, short-term remissions are much more likely as compared to patients with solid tumors [6]. This then creates significant difficulty in gauging when to involve the PC team or when to engage hospice care. Because of these prognostic challenges of clinical judgment, several studies have emphasized the need to move away from referrals to PC based on prognosis or life expectancy and move toward basing the PC intervention on individual and family/caregiver needs [3]. Importantly, a significant clinical or provider barrier is the general misperception of PC services being equated or misconstrued as end-of-life care and, therefore, often thought of being implemented only when the patient is dying, nearly dying, or decompensating and deteriorating [3].

In terms of barriers from the patient perception of PC, patients with HMs undergoing BMT may often prefer to focus discussions with their providers on curative rather than palliative goals. As discussed later on, there are significant cultural influences on the discussion of death and dying combined with misperceptions of PC that may further contribute to patient barriers toward implementing PC early on in their care. Other perspectives discussed by studies include the view that BMT patients perceive their treatment as high-risk and, thus, that the nature of the treatment leads to expectations of suffering and symptom burden, so some patients may not actively seek or feel the need to alleviate themselves from experiencing symptoms [6].

Finally, several logistic and administrative barriers toward early PC interventions in this patient population center on resource constraints and the complexity of care organization/policy. For BMT patients specifically, one key PC treatment to relieve certain symptoms is at-home transfusion support, which can be incredibly difficult to perform and coordinate logistically [2]. As this often conflicts with policy and standards set in place for hospice care, this is a clear example of logistical and policy challenges to provide the full range of palliative support necessary for implementing early PC interventions for BMT patients [2]. Therefore, these numerous barriers emphasize the need for clearly defining and deciphering common perceptions and misperceptions of early PC interventions that may aid in the development of standardized care models to improve the quality of life and care for patients undergoing BMT.

In PC, there are significant disparities between Whites and racial/ethnic minorities. The lack of literature on the impact of culture on patients’ understanding of disease likely promotes this disparity [8]. People of cultural minorities experience barriers to PC to the extent that minorities are more prone to receiving treatment that does not coincide with their care goals [9]. A study by Mack et al. further demonstrates this misalignment whereby despite having their wishes documented, African Americans are less likely than their White counterparts to have their care goals met [10]. Regional differences in access to PC are another barrier minorities face. Dumanovsky et al. report this to be the case for minorities in the Southern United States, where inadequate access to PC may result from a regional lack of large hospitals, which are more likely to offer palliative services [11]. Also limited by geography is the ability to offer pain management, a core aspect of PC. One study demonstrates that African Americans lack accessibility to pain medication because they tend to live in areas where pharmacies do not supply opioids [12]. Despite these physical barriers and disparities, an improved understanding of the influence of culture in PC will likely bridge the gap between the care received by racial/ethnic minorities relative to Whites.

Cultural aspects

Culture is an important influence on patients’ preferences with respect to the processes, types of care providers, and extent of care they would like to receive during their recovery [13]. One process includes the extent to which patients want to be informed about their condition [14,15]. Certain Native American cultures, for example, prefer not to speak about death, limiting their access to PC, and widening the disparity relative to Whites [16]. Another key process includes shared decision-making. In the United States healthcare setting, maintaining respect for patient autonomy is at the forefront of this discussion. However, persons of different backgrounds and beliefs may not value autonomy highly during clinical decision-making [17]. Culture also influences whom patients desire to be named as their care providers. For instance, African Americans more often emphasize the involvement of community leaders during end-of-life planning over more formal discussions with their medical providers [18]. This likely stems from the long history faced by African Americans of institutional abuse through slavery and mistreatment during research [13]. The extent of care desired is another aspect that varies by cultural group. Studies have found that relative to Whites, African Americans and Hispanics opt for more intensive end-of-life interventions [19]. For example, African Americans and Hispanics are more likely to experience hospitalization, ICU admission, and advanced life-sustaining therapies (CPR, intubation, mechanical ventilation, etc.) near the end of life as compared to Whites [19]. Cain et al. note that although evidence-based generalizations may help with a foundation of knowledge of different cultural/ethnic groups, it is important to still ask and discuss with patients about their PC preferences [13]. There is no single pattern of preferences that each cultural group follows, but rather there are in-group variations [19]. Because culture has a broad influence on patients' preferences of processes, care providers, and breadth of treatment, further study is needed on the integration of culture into PC and its role in influencing perceptions and misperceptions of PC.

Current care models

In a review of the current literature assessing the need for PC in HM and BMT patients, Epstein et al. find that patients with HMs will likely benefit from increased access to PC [20]. However, the study also notes the need for more work demonstrating the effects of various forms of PC delivery and implementation in the HM population. Currently, there has only been one randomized trial, by El-Jawahri et al., demonstrating the incorporation of PC into HM care [21]. Quality of life and symptom control including management of psychological distress were significantly improved in this model that involved biweekly in-patient consults by PC-trained physicians and nurse practitioners over a one-month period. Another study attempted to maximize the distribution of limited PC services to the greatest number of critical HM patients by utilizing PC “office hours” [22]. By offering this service within the hospital, hematologists were able to receive PC support without requiring a formal PC consultation. Barriers to the entry of PC into hematology have been discussed earlier in our review but include the misperception by hematologists that PC equates to end-of-life care [23]. LeBlanc et al. also note that hematologists are less likely to manage patients with others, such as PC physicians, due to the longitudinal nature of HM care, which contributes to more possessive physician behavior toward patients. Many recommendations on the integration of PC into the management of patients with HM being treated with BMT are extrapolated from the positive effects PC has demonstrated in the care of patients with non-HMs. Well-studied care models from the non-HM aspect include establishing PC services in cancer centers [24,25] and improving patient-centered communication and PC techniques in the training of oncologists [26,27]. Further study is needed in demonstrating the efficacy of various PC models in this subpopulation of HM patients undergoing BMT.

Potential niches

Relative to patients with metastatic non-HMs or solid tumors, patients with HMs experience a similar number of symptoms with those symptoms causing an equal degree of distress [28]. Inadequate management of this distress can negatively impact treatment outcomes and worsen quality of life [29]. Despite these findings, the role of PC in this population has been limited. Psychological symptoms, such as anxiety and feelings of sadness, were reported in over 75% of patients in the Manitta et al. study, with these symptoms presenting more severely in patients with HMs as compared to patients with metastatic non-HMs [28]. This finding highlights the potential for PC as a mechanism to address the specific and more prominent psychosocial needs of patients with HMs.

Another potential niche for PC in patients with HMs undergoing BMT comes in the form of improving prognostic understanding. Awareness of one’s prognosis is especially important in the decision-making process of patients with HMs who must consider significant interventions such as chemotherapy and BMT [30]. El-Jawahri et al. describe that over 70% of both patients with HMs and their caregivers hold inappropriately optimistic views of their prognosis relative to that of their oncologist. However, this study also notes that when patients with HMs do maintain a prognostic outlook that is in line with their oncologist’s views, they are more likely to have a worse symptom profile, experiencing more fatigue and depressive episodes. Temel et al. found in a study on a non-HM that early implementation of PC improved prognostic understanding [25]. This supports the proposition that PC serves a key, adjunctive role to standard oncological care in the management of difficult cases and should be explored further in patients with HMs undergoing BMT [23].

## Conclusions

Patients living with HMs and undergoing BMT are significantly more likely to receive aggressive care toward the end of life and are more likely to have reduced quality of life near the end of life as compared to patients with solid tumors. These findings are associated with the severity of treatment-related complications of a BMT, which contributes to the symptomatology and aggressive treatments these patients experience. Despite the considerable symptom burden, patients with HMs undergoing BMT are also less likely to receive specialty PC referrals and are most often referred within the final months before the end of life. Further efforts should be aimed at improving the understanding of perceptions from both patient and provider perspectives regarding PC interventions to develop a care model integrating PC services with the care of patients with HM undergoing BMT. Several studies have identified a number of barriers affecting the implementation of PC referrals for this specific patient population. Some of the most commonly encountered clinical barriers for both provider and patient include misperceptions of equating PC to end-of-life care, overestimation or uncertainty of patient prognostication leading to challenges on deciding when to implement PC interventions, hesitancy to transition from curative to palliative goals of care, and logistical and organizational policy challenges of incorporating certain palliative treatments into practice. Similarly, social barriers can be created when failing to incorporate patient culture into care goals, with culture influencing key aspects of how patients view life, death, and treatment at the end of life. Due to the aforementioned barriers to the practice of PC in this population of patients, few studies have examined the efficacy of different care models of PC in patients with HM undergoing BMT. Despite the still-developing practice of PC in this setting, potential niches for PC include decreasing symptom burden, notably psychological ones, as well as improving patient’s prognostic understanding. The consideration of important clinical and cultural PC barriers, lack of existing standardized PC models, and pursuit of potential PC niches highlights the need for a better understanding of the perceptions and misperceptions of early PC interventions in patients with HMs undergoing BMT to provide meaningful insight into improving the quality of life and maximizing compassionate care for this resilient patient population. Improving research and study on the implementation of specific early PC interventions and enhancing the perception of both providers and patients toward the importance and benefits of PC use are warranted to continue improving the care of patients with HMs undergoing BMT. 
